# Chronic treatment with Tempol during acquisition or withdrawal from CPP abolishes the expression of cocaine reward and diminishes oxidative damage

**DOI:** 10.1038/s41598-017-11511-7

**Published:** 2017-09-11

**Authors:** Tehila Beiser, Ran Numa, Ron Kohen, Rami Yaka

**Affiliations:** Institute for Drug Research (IDR), School of Pharmacy, Faculty of Medicine, The Hebrew University of Jerusalem, Jerusalem, 91120 Israel

## Abstract

In previous studies, we reported that pretreatment with the antioxidant Tempol attenuated the development and expression of cocaine-induced psychomotor sensitization in rats and diminished cocaine-induced oxidative stress (OS) in the prefrontal cortex (PFC) and nucleus accumbens (NAc), suggesting a potential role for Tempol in interfering with cocaine-related psychomotor sensitization. The aim of the current study was to examine the role of Tempol in reward and reinforcement using the conditioned place preference (CPP) paradigm. We found that administration of Tempol during the conditioning session abolished the expression of cocaine-induced CPP. We also found that OS was significantly elevated following the establishment of CPP, and that cocaine-induced OS was significantly diminished by pretreatment with Tempol during conditioning. Furthermore, we found that repeated, but not single, administration of Tempol for seven days during withdrawal from CPP resulted in significant attenuation in the expression of CPP. Moreover, Tempol did not affect the expression of food reward. Taken together, these findings provide evidence for the involvement of Tempol in regulating cocaine rewarding properties without affecting natural rewards. Since Tempol was found to be effective in reducing OS and expression of CPP following withdrawal, it may be a potential treatment for cocaine addiction.

## Introduction

Cocaine is harmful to almost all organs. Aside from addiction, it may lead to cardiotoxicity, hepatotoxicity, nephrotoxicity, and even to retinal damage^1–4^. In the central nervous system (CNS), cocaine use may result in neurotoxicity, neuroinflammation^5–8^ and neurodegeneration^9, 10^. Evidence associating the harmful outcome of cocaine toxicity with oxidative stress (OS) has accumulated during the last decade, and it is believed to play a main role in the negative consequences of cocaine intake. OS is defined as imbalance between the formation of reactive oxygen species (ROS) or reactive nitrogen species (RNS), and endogenic compensative antioxidant system activity. The CNS is the most vulnerable to OS, because of its high oxygen consumption and high levels of polyunsaturated fatty acids.

Cocaine is a very potent psychostimulant. Its stimulating and euphoric effects result from an increase in dopamine (DA) and other catecholamine levels in the mesolimbic DA system caused mainly by blockage of their reuptake into dopaminergic terminals. As a result of cocaine intake, its toxic oxidative metabolites as well as DA metabolites are increased^5^, among them toxic products of DA autoxidation^11^. It has been demonstrated that cocaine induces OS in several brain areas by increasing hydrogen peroxide and nitric oxide (NO) synthase (NOS)^12, 13^. Cocaine-induced OS leads to oxidation and apoptosis^14, 15^, and elevates lipid peroxidation metabolites^13, 16, 17^. Cocaine administration may result not only in ROS and RNS elevation, but also in a depletion of the enzymatic components of the endogenous antioxidant defense system, including catalase (CAT), superoxide-dismutase (SOD), and glutathione-peroxidase^18–20^. Moreover, cocaine exposure has also been linked to reduction of non-enzymatic antioxidant levels^20^, including reduced glutathione (GSH) or vitamin E, resulting in elevated ROS and damaged brain function^21, 22^. Thus, the need for a suitable agent that will diminish cocaine-induced OS has been raised. A member of the nitroxide family, Tempol (4-hydroxy-2,2,6,6 tetramethylpiperidine-N-oxyl), is a unique antioxidant with several advantages, such as membrane permeability^23, 24^, penetration of the blood brain barrier^6, 25^, and most importantly, ability to mimic SOD activity^26^. Together, these properties render Tempol a suitable candidate to provide neuroprotection against cocaine toxicity.

Previously, we reported that pretreatment with the antioxidant Tempol diminished cocaine-induced oxidative damage *in vitro* in the PC12 cell line, *ex vivo* in slices containing the nucleus accumbens (NAc) and prefrontal cortex (PFC), and *in vivo* following acute and chronic cocaine exposure^16, 27^. Subsequently, Tempol was shown to attenuate both cocaine- and methamphetamine-induced OS^28, 29^. Importantly, we demonstrated that pretreatment with Tempol attenuated the development and expression of cocaine psychomotor sensitization in rats via decreased OS in the NAc and PFC^16^. Recently, it was found that microinjections of Tempol to the NAc shell blocked morphine conditioned place preference (CPP) through the mGluR5 signaling pathway^30^, emphasizing the important role of OS induced by other drugs of abuse.

Since we have previously demonstrated that Tempol prevents cocaine-induced psychomotor sensitization, in the current study we aimed to evaluate the effect of Tempol on cocaine reward using the CPP paradigm. We tested the effect of Tempol on the acquisition of cocaine reward when administered as a pretreatment to cocaine during CPP conditioning. We further tested the ability of Tempol to interfere with the expression of cocaine CPP when administered during withdrawal following CPP.

## Materials and Methods

### Animals

Sprauge Dawly male rats (Harlan Laboratories, Jerusalem) weighing 90–100 gr were kept in groups of four with food and water available ad libitum. A 12-hour light/dark cycle was used with the lights on at 7:00 A.M. All experiments were performed according to the guidelines of the Institutional Animal Care Committee of the Hebrew University (Jerusalem, Israel). The joint ethics committee (IACUC) of the Hebrew University and Hadassah Medical Center approved the study protocol for animal welfare. The Hebrew University is an AAALAC International accredited institute. The experiments were designed and performed in a manner that aimed to minimize the discomfort of the animals.

### Conditioned place preference (CPP)

The CPP apparatus (Med Associates) consists of two visually distinct conditioning compartments. One contains white-colored walls and wire mesh flooring (28 cm × 21 cm) while the other has black-colored walls and steel rod flooring (28 cm × 21 cm). The compartments are connected by a smaller center compartment (12 cm × 21 cm). Infrared beams located at the bottom of the wall allow assessment of animal preference for each compartment. CPP experiments were carried out at predefined times of day. Following a 3 day acclimation, a biased CPP design was conducted as follows: the animals were placed in the central gray compartment for 5 min and then they were free to explore all three compartments for 15 min. The time spent in each compartment was analyzed by automated software and the results were used to determine the initial preference. The least preferred compartment for each subject was then assigned to be the drug-paired compartment. The conditioning period began one day following the habituation session. Cocaine or saline injections were given each day. The animals received four saline (1 ml/kg, i.p.) and four cocaine (15 mg/kg, i.p.) injections on alternating days and were confined to the allocated compartment for a 15 min period. Thus, a total of 8 days of training were conducted. To evaluate the establishment of cocaine-induced CPP, we tested the animals one day following the last conditioning day, unless otherwise stated in the text. Each animal was placed in the central compartment for 5 min followed by a 15 min period of free access to all compartments. CPP score was defined as the percentage determined by: 100*(time spent in drug-paired chamber − time spent in saline-paired chamber)/ (time spent in drug-paired chamber + time spent in saline-paired chamber). To test whether Tempol has aversive or reinforcing properties by itself, we conducted CPP procedure identical to cocaine–CPP as described above, using Tempol (200 mg/kg) as the pairing agent.

For food-induced CPP, animals were deprived of food during all the conditioning period in order to lower their body weights by 10–15%. 10 min prior to the conditioning sessions, two groups of animals received daily i.p. injection of Tempol (200 mg/kg) or saline (1 ml/kg) 10 min prior to the exposure to the CHEETOS®-containing chamber. On alternating days during the conditioning period, the animals were placed in the non-preferred chamber with CHEETOS®, while on the next day an empty dish was introduced at the opposite chamber. On the test day, the same procedure was used as in the case of cocaine induced CPP.

When Tempol was administered during withdrawal, the standard cocaine CPP protocol was conducted. One day after completion of conditioning to cocaine, half of the rats from each treatment group received daily injections of Tempol (200 mg/kg) for seven days, while the other half received daily injections of saline (1 ml/kg). On the 7th day, the CPP test was performed as described above.

### Assessment of lipid peroxidation

24 hours following cocaine-induced CPP, animals were euthanized and their brains were immediately removed. PFC and NAc were microdissected, homogenized and analyzed for OS. The extent of lipid peroxidation in animal brain tissue was estimated colorimetrically using the thiobarbituric acid reactive substances (TBARS) method as we have previously described^16^. Briefly, 0.1 ml aliquots of tissue homogenates were treated with 2 ml of (thiobarbituric acid (TBA)–TCA–HCl 1:1:1 ratio) reagent containing: thiobarbituric acid 0.37% (Sigma, St. Louis, USA) - TCA 15% (Baker, NJ, USA) - HCI 0.25 N (1:1:1 ratio). Samples were heated to 95 °C for 30 min, cooled on ice and centrifuged (12,000 rpm, 10 min). The clear supernatant fluids were measured at 535 nm with a microplate reader (Bio-TEK ELx, Winooski, VT, USA). A malonaldehyde bis (dimethyl acetal) (Sigma, St. Louis, USA) standard curve was prepared under the same experimental conditions. Calculations were made on the basis of standard curves and presented as µmol malondialdehyde (MDA)/g protein.

### Evaluation of nitrites

24 hours following cocaine-induced CPP, animals were euthanized and their brains were immediately removed. PFC and NAc were microdissected, homogenized, and analyzed for OS. Tissue homogenates were interacted (1:1) with Griess reagent consisting of: 0.1% naphthylethylenediamine dihydrochloride (Sigma, St. Louis, USA), 1% sulfanilamide (Sigma), and 2% H_3_PO_4_. Fifteen minutes later, the absorbance of the clear supernatant fluid was measured at a wavelength of 540 nm with a microplate reader (Bio- TEK ELx, Winooski, VT, USA). A sodium nitrite (Sigma, St. Louis, USA) standard curve was prepared under the same experimental conditions. Results are presented as µmol sodium nitrites/g protein^31^.

### Statistical analysis

Effect of treatment on acquisition of cocaine-induced CPP was assessed with one-way ANOVA followed by *posthoc* Tukey HSD tests when significant. To evaluate significance of CPP, one sample *t*-test was used. For multiple comparisons between groups, we used one-way ANOVA followed by Tukey HSD *post hoc* tests. Data are presented as mean ± S.E.M. The accepted value of significance for all tests was set at *p* < 0.05 and indicated by asterisks in the figures. All group sizes and significant differences are reported in the figure legends. The statistical analysis was performed using IBM SPSS Statistics for Windows, Version 21.0. Armonk, NY.

## Results

### Tempol abolished the acquisition of cocaine CPP

**Figure 1:**
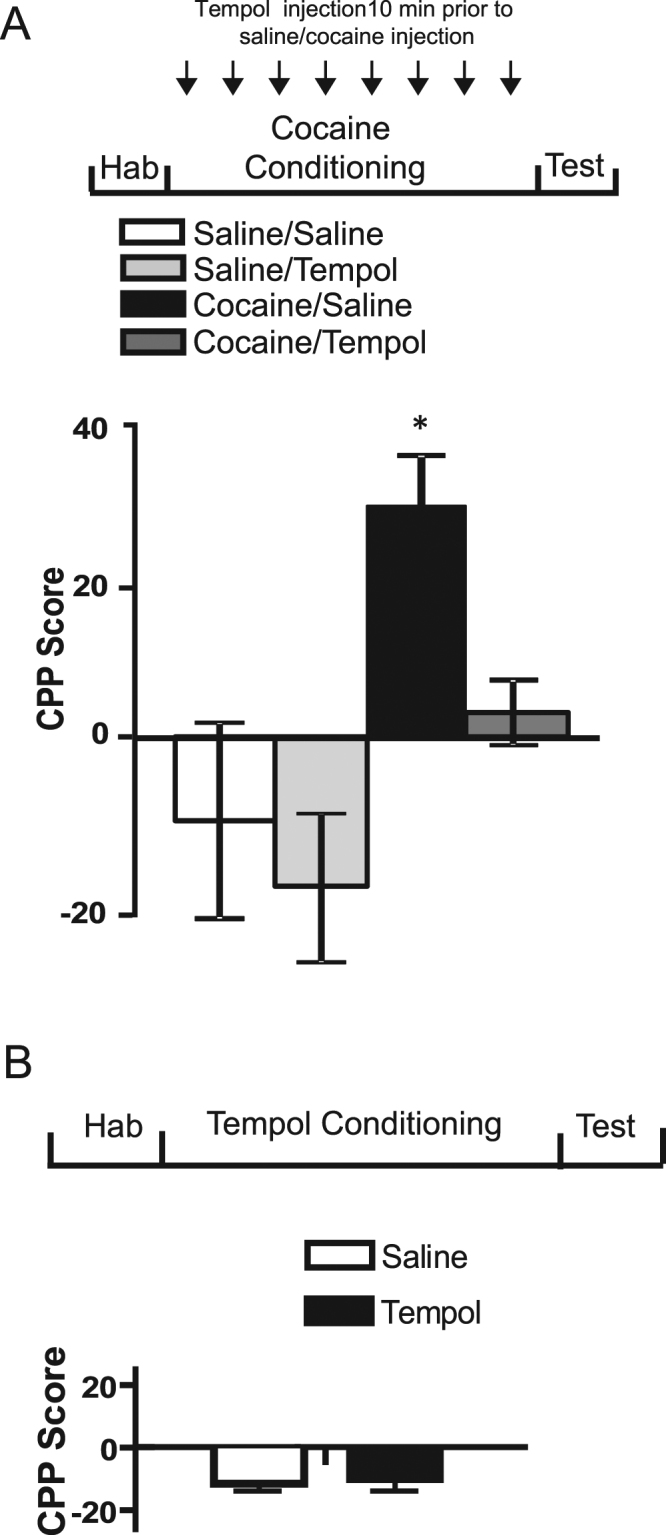
Tempol administered prior to cocaine during conditioning abolished cocaine- induced CPP. (**A**) Rats were randomly assigned to four groups for CPP study. All groups were given i.p. injections of either saline (1 ml/kg) or Tempol (200 mg/kg) 10 min before the conditioning sessions. Values are expressed as mean CPP score ± S.E.M. (*n* = 7, 8, 13 and 14 rats for the saline/saline, Tempol/saline, saline/cocaine, and Tempol/cocaine groups, respectively). Hab = Habituation, *Differ from all other groups (*p* < 0.05). (**B**) CPP protocol was conducted employing Tempol as the paired agent. Tempol (200 mg/kg) and saline (1 ml/kg) were given on alternate days for eight conditioning sessions. Values represent mean CPP score ± S.E.M. (*n* = 8 rats per group) on the test day.

We first evaluated whether pretreatment with Tempol affects the acquisition of CPP induced by cocaine. As shown in Fig. 1A, the administration of Tempol (200 mg/kg), a dose that did not affect acute locomotor response^16^ 10 min prior to each cocaine or saline injection during each conditioning session, completely abolished the acquisition of cocaine-induced CPP. In the test, a significant effect of treatment was revealed by one-way ANOVA (*F*_3,39__=_ 8.54, *p* < 0.01). The Tempol-cocaine group did not demonstrate CPP, and did not differ from the groups that had not been treated with cocaine (saline-saline and Tempol-saline groups). Significant preference was found only in the saline-cocaine group (one sample *t*-test: *t* = 4.21, *p* < 0.01). Post-hoc Tuckey HSD tests revealed that this group differed from other groups (*p* < 0.05). These results suggest that pretreatment with Tempol during conditioning abolishes the expression of cocaine-CPP.

The possibility that Tempol itself might induce preference or aversion in the CPP paradigm was also examined. The typical CPP protocol was conducted, except that Tempol was employed as the paired agent instead of cocaine. Figure 1B shows that rats conditioned with Tempol (200 mg/kg) did not show any significant changes in CPP score compared to the control saline group (treatment effect: *F*_1,28_ = 0.15, *p* = 0.70), indicating that Tempol administration had no rewarding or aversive properties.

### Tempol does not attenuate food-induced CPP

**Figure 2:**
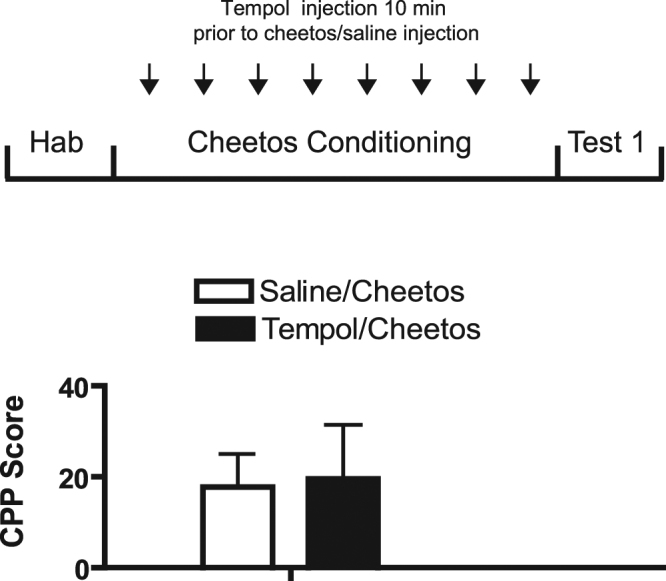
Tempol does not attenuate food-induced CPP. Following conditioning to CHEETOS®, CPP scores were estimated for the Tempol and saline groups. On alternating days, rats were placed in one chamber with CHEETOS® pellets and in the opposite chamber with an empty dish. They received daily i.p. injection of either Tempol (200 mg/kg) or saline (1 ml/kg). Hab = Habituation. Values represent mean CPP score ± S.E.M. (*n* = 8 rats per group).

Since Tempol blocked the acquisition of cocaine-induced CPP (Fig. 1A), it was also important to evaluate whether it could affect the preference for natural rewards. Therefore, we tested several natural food reinforcers and found that rats were attracted to CHEETOS® snack (CHEETOS®). Accordingly, rats were conditioned to CHEETOS® as described in the Methods section. CHEETOS® induced CPP, though to a lesser degree than cocaine. However, as shown in Fig. 2, Tempol had no effect on food-induced CPP. The CPP scores of rats that had been treated with Tempol did not show any significant differences from rats that received saline, as confirmed by a one-way ANOVA (treatment effect: *F*_1,28_ = 0.09, *p* = 0.76). The results indicate that although Tempol has a distinctive effect on cocaine reward, it does not interfere with natural rewards such as food.

### Tempol during the conditioning period reduces cocaine-induced OS

**Figure 3:**
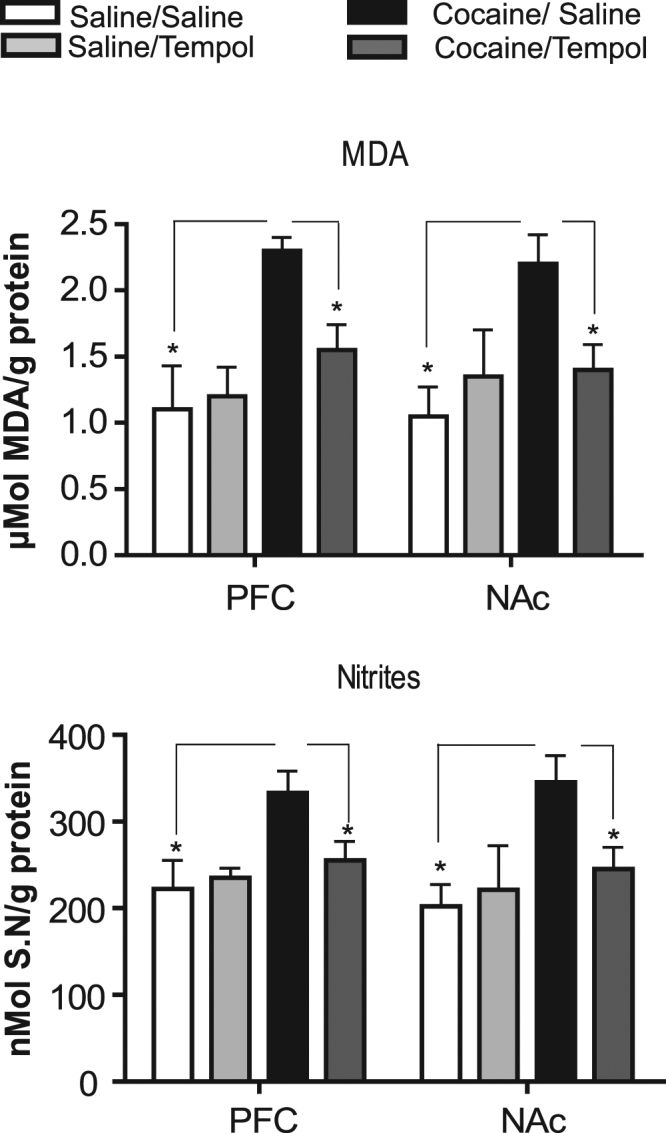
Cocaine CPP induces oxidative stress (OS) during or following conditioning, which can be restored by Tempol. Animals were treated as in Fig. 1A. Lipid peroxidation (MDA) and nitrite levels in PFC and NAc samples were determined following CPP. Values are expressed as mean ± S.E.M. (*n* = 8 rats per group). *Differ from saline/cocaine group (*p* < 0.05).

To examine whether cocaine induces oxidative damage during cocaine-CPP and whether Tempol can alter these changes, OS was evaluated following cocaine-induced CPP. The results presented in Fig. 3 demonstrate a significant effect of treatment on levels of MDA (µmol MDA/g protein), a lipid peroxidation indicator, as confirmed by one-way ANOVA both in the PFC (*F*_3,28_ = 5.81, *p* < 0.05) and in the NAc (*F*_3,28_ = 5.33, *p* < 0.05) (Fig. 3). Similar results were obtained with respect to nitric oxide radical metabolite levels (indicated by nitrite levels, nmol sodium nitrite/g protein, Fig. 3) both in the PFC (*F*_3,28_ = 4.26, *p* < 0.05) and NAc (*F*_3,28_ = 4.16, *p* < 0.05). Administration of Tempol prior to cocaine induced a significant decline in MDA levels as compared with saline/cocaine treatment in both the PFC (*p* < 0.01) and NAc (*p* < 0.05), such that MDA values in the Tempol/cocaine group did not differ from those in the saline/saline group in both the PFC (*p* > 0.05) and NAc (*p* > 0.05). Nitrite levels following Tempol/cocaine treatment showed a significant decrease relative to saline/cocaine treatment in both the PFC (*p* < 0.05) and NAc (*p* < 0.05) and did not differ from the values observed after saline/saline treatment both in the PFC (*p* > 0.05) and NAc (*p* > 0.05). These results suggest that Tempol given during conditioning significantly reduced cocaine-induced OS.

### Tempol administered during withdrawal abolished the expression of CPP

Having found that OS levels were increased following cocaine conditioning, we hypothesized that administration of Tempol during withdrawal would negatively affect cocaine-CPP. We first examined whether a single injection of Tempol one day following CPP, will interfere with the expression of CPP.

**Figure 4:**
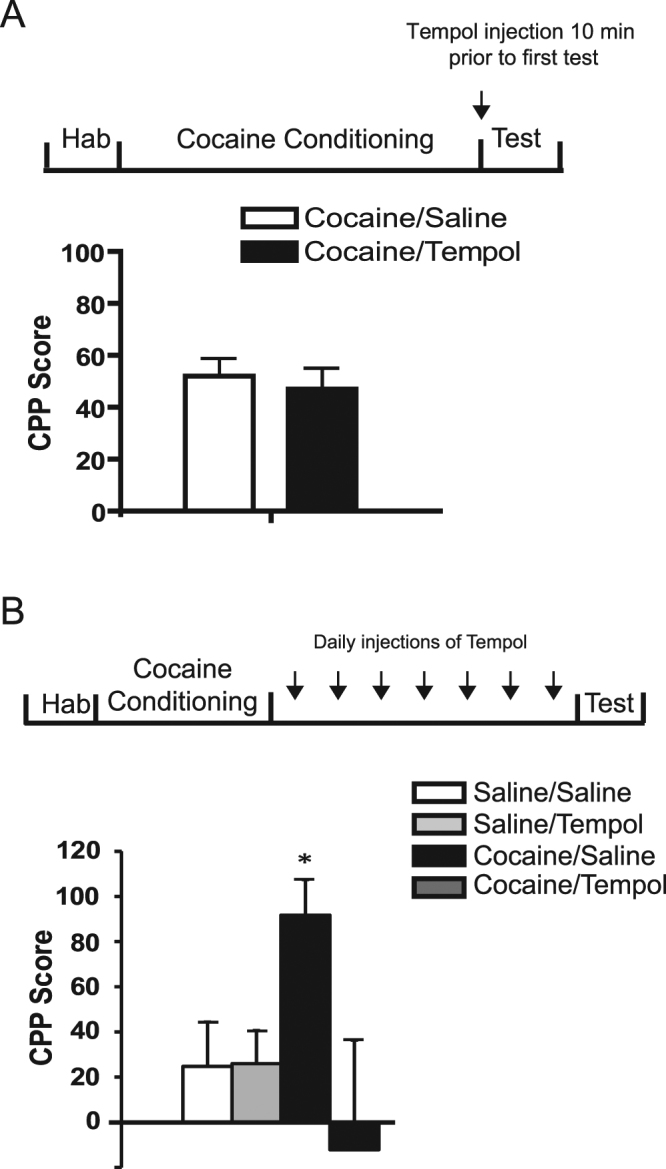
Tempol administered during withdrawal abolished the expression of CPP. (**A**) Following a standard cocaine CPP paradigm, animals received a single injection of either Tempol (200 mg/kg) or saline (1 ml/kg) immediately prior to testing. Values represent mean CPP score ± S.E.M. (*n* = 8 rats per group). (**B**) Rats were randomly assigned to two groups for CPP study. One day after conditioning, both groups were administered with daily injections of either Tempol (200 mg/kg) or saline (1 ml/kg) for 7 days in their home-cage. The test was performed on the 7^th^ day. Hab = Habituation. (*n* = 10 for cocaine groups and *n* = 9 for saline groups). *Differ from all other groups (*p* < 0.05).

Therefore, a typical cocaine-induced CPP protocol was performed, except that on the test day, rats were divided into two groups and then received a single injection of either Tempol (200 mg/kg) or saline (1 ml/kg) immediately prior to testing. Figure 4A shows that the expression of CPP was evident and that the CPP scores did not reveal any significant difference between the two groups (treatment effect: *F*_1,28_ = 0.99, *p* = 0.32). These results suggest that a single injection of Tempol prior to the test is not sufficient to interfere with the behavioral outcome of CPP. Moreover, it demonstrates that Tempol has no effect on the state of the animal at the time of the test, ruling out the possibility that Tempol interferes with the expression of CPP regardless of previous experience during cocaine conditioning. Thus, we hypothesized that chronic treatment with Tempol will have a stronger impact on the expression of CPP. Therefore, we performed the CPP protocol as above described. One day following the CPP protocol, half of the rats from each treatment group received daily injections of Tempol (200 mg/kg) for seven days, while the other half received daily injections of saline (1 ml/kg) for seven days, including the test day. On the 7^th^ day, the CPP test was performed. Figure 4B clearly demonstrate that chronic treatment with Tempol (but not acute single exposure) abolished the expression of cocaine CPP. A significant effect of treatment was found in the cocaine/Tempol group (*F*_3,34_ = 3.05, *p* < 0.05). Preference for the cocaine-paired chamber was observed only in rats treated with saline (cocaine/saline) as compared to the rats treated with Tempol (cocaine/Tempol) (one sample *t*-test: *p* < 0.01). Thus, daily administration of Tempol following conditioning completely abolished cocaine-induced CPP.

## Discussion

Our previous findings suggest that Tempol exerts a protective effect against cocaine toxicity by diminishing cocaine-induced OS in the NAc and PFC, not only when given prior to acute exposure to cocaine but also when given prior to repeated cocaine administrations^16^. In addition, we found that Tempol have a protective effect by attenuating the development and expression of cocaine psychomotor sensitization^16^. These results led us to hypothesize that the involvement of Tempol in reducing cocaine-induced sensitization is not unique to this type of cocaine-induced behavior and that Tempol may have a beneficial effect on other cocaine-related addictive properties, such as the rewarding effect of cocaine.

Based on this hypothesis, the present study shows that Tempol is indeed involved in regulating cocaine reward as measured by the CPP model in rats. The results indicate that administration of Tempol immediately prior to each cocaine conditioning session abolished the preference for cocaine, suggesting that Tempol impairs the acquisition of cocaine CPP. The substantial behavioral changes in the Tempol-administered animals were correlated with attenuation of cocaine-induced oxidative damage in their NAc and PFC, as estimated by measurement of lipid peroxidation metabolite levels and nitrite levels. These results suggest that the beneficial contribution of Tempol in reversing cocaine reward is mainly due to its antioxidant properties.

We demonstrated that Tempol impaired cocaine-conditioned reward and prevented OS when administered prior to cocaine. The half-life of Tempol following acute injection is expected to last more than 10 min^32, 33^, which was the time difference between Tempol and cocaine injections in the current study. Thus, Tempol was still in circulation when cocaine was injected. However, in order to use Tempol to prevent cocaine-induced behaviors after withdrawal from cocaine, we tested its ability to interfere with reward processing following acquisition. We found that a single injection of Tempol following cocaine conditioning and prior to the test was not sufficient to impair cocaine CPP. Since a single injection was not sufficient, repeated daily injections of Tempol for seven days following conditioning were performed and they indeed abolished the expression of CPP. These results suggest that antioxidants such as Tempol are effective in interfering with reward processing and associative learning even when they are not paired with drug exposure and given during abstinence. Similarly, abolishment of morphine CPP was found when Tempol was microinjected to the NAc shell following morphine conditioning and immediately prior to CPP tests^30^. Together, these findings suggest that production of ROS is critically involved in the establishment of CPP and may be manipulated using antioxidants. Moreover, these studies clearly suggest that antioxidants given after establishment of addictive behaviors can serve as a new approach to drug therapy. It should be noted, however, that when Tempol was given in a single dose following conditioning to cocaine, it failed to attenuate expression of CPP, suggesting that repeated antioxidant administration is required.

Natural rewards such as food can also motivate animals and induce CPP. The necessity of the anti-oxidative properties of Tempol in reversing drug-induced behaviors was substantiated by the absence of the effect of Tempol on snack-food CPP. Palatable food consumption increases DA levels in the reward system^34^ and therefore we assume that as a result, the level of ROS is elevated, but to a much lower extent as compared to drugs of abuse. Our results are in line with a previous study showing that systemic administration of ROS scavengers including Tempol attenuated METH-induced locomotor activity and self-administration without affecting food consumption^29^. Furthermore, this study demonstrated an increase of OS in the NAc of METH self-administering rats. Taken together, these results suggest that Tempol and non-specific scavengers could diminish cocaine- or METH-induced ROS formation specifically, without affecting food intake.

One of the main questions raised by the current study deal with the molecular mechanisms underlies the involvement of OS in reward processing and addiction. It is well accepted that the molecular machinery that sub serve learning and memory is “hijacked” by drugs of abuse^35^. Among the first direct evidence that link cocaine action to synaptic changes was the findings that a single cocaine injection resulted in an increase of AMPA/NMDA receptors current ratio in VTA DA neurons, an indicator for *in vivo* like LTP^36^. Subsequently, it was shown that repeated injections of cocaine or other drugs of abuse elevate this ratio during the period of drug exposure^37^ and following withdrawal^38^. Furthermore, it was demonstrated that following withdrawal from repeated cocaine injections or self-administration, the levels of GluA1 of AMPA receptor (AMPAR) were increased in the NAc^39, 40^. It was therefore suggested that these changes are responsible for cue-induced reinstatement in several behavioral models^39, 40^. We have previously shown that withdrawal from repeated cocaine injections increases NMDA receptors (NMDAR) redistribution the synaptic membrane which in turn activates the MAPK signaling pathway that eventually leads to an increase in synaptic GluA1 in the NAc^40^. Taken together, these results suggest that GluA1 is one of the main targets for cocaine-induced neuroadaptations. NMDAR stimulation also leads to neuronal nitric oxide synthase (nNOS) activation, and thus to NO formation^41^. A direct evidence linking between OS and synaptic changes was demonstrated when NO formation was found to induce GluA1 S- nitrosylation that was shown to prevent AMPAR binding to the AP2 protein complex of the endocytotic machinery^42^. As a result, the increase of synaptic AMPAR expression and conductance was reported. Based on these studies, it is tempting to speculate that OS induced alterations in the function of synaptic proteins, such as the NMDAR or AMPAR, that play a pivotal role in the development and expression of cocaine addiction and antioxidants such as Tempol can reverse the oxidative damage and therefore can attenuate cocaine-induced behaviors.

It is well established that increased DA transmission within the reward system is associated with the behavioral response to cocaine. It was suggested that following cocaine self-administration, the accumulation of dopamine and its metabolites in the brain results in increased production of ROS (e.g. dopamine quinone and 6-hydroxydopamine)^11, 43^. It was also proposed that enhanced ROS production might occur by oxidized metabolites of cocaine (e.g. norcocaine derivatives), throughout redox cycling involving several possible electron transfer agents^5^, or by brain microsomes^44^. Recently, it was shown that both cocaine and methamphetamine increase oxidative stress, and two ROS scavengers, Tempol and PBN, significantly attenuated cocaine- or METH-self administration and significantly reduced the enhancement of DA release in the NAc of self-administrating rats^28, 29^. Therefore, it is likely to assume that the abolishment of cocaine conditioned reward by Tempol is partly due to the reduction of DA release in the NAc, and as a consequence decreased damage to key proteins mediating the reinforcement properties of cocaine.

In conclusion, our study provided evidence for the interaction between Tempol and cocaine administration and demonstrated that Tempol inhibited the acquisition and expression of cocaine CPP, suggesting that Tempol regulates the rewarding properties of cocaine. This study, which increases our understanding of the pharmacological function of Tempol and its relationship with cocaine neuroadaptations in the CNS, may implies Tempol as a potential target for pharmacological treatment of cocaine addiction.

## References

[1] Pendergraft WF, Herlitz LC, Thornley-Brown D, Rosner M, Niles JL (2014). Nephrotoxic Effects of Common and Emerging Drugs of Abuse. Clin. J. Am. Soc. Nephrol..

[2] Sánchez-Villarejo MV (2014). Chronic Cocaine Effects in Retinal Metabolism and Electrophysiology: Treatment with Topiramate. Curr. Eye Res..

[3] Stankowski RV, Kloner RA, Rezkalla SH (2014). Cardiovascular consequences of cocaine use. Trends Cardiovasc Med.

[4] Graziani, M. *et al*. Cardiovascular and hepatic toxicity of cocaine: Potential beneficial effects of modulators of oxidative stress. *Oxid. Med. Cell. Longev*. **2016**, (2016). 10.1155/2016/8408479 PMC4707355 26823954

[5] Kovacic P (2005). Role of oxidative metabolites of cocaine in toxicity and addiction: Oxidative stress and electron transfer. Med. Hypotheses.

[6] Liang Q (2005). Neuroprotective effects of TEMPOL in central and peripheral nervous system models of Parkinson’s disease. Biochem. Pharmacol..

[7] Kousik SM, Napier TC, Carvey PM (2012). The effects of psychostimulant drugs on blood brain barrier function and neuroinflammation. *Front*. Pharmacol..

[8] Thrash-Williams B (2016). Methamphetamine-induced dopaminergic toxicity prevented owing to the neuroprotective effects of salicylic acid. Life Sci..

[9] Cunha-Oliveira T, Rego aC, Oliveira CR (2013). Oxidative Stress and Drugs of Abuse: An Update. Mini. Rev. Org. Chem..

[10] Lax E (2013). Neurodegeneration of lateral habenula efferent fibers after intermittent cocaine administration: Implications for deep brain stimulation. Neuropharmacology.

[11] Smythies J, Galzigna L (1998). The oxidative metabolism of catecholamines in the brain: A review. Biochim. Biophys. Acta - Gen. Subj..

[12] Dietrich JB (2005). Acute or repeated cocaine administration generates reactive oxygen species and induces antioxidant enzyme activity in dopaminergic rat brain structures. Neuropharmacology.

[13] Bashkatova V, Meunier J, Vanin A, Maurice T (2006). Nitric oxide and oxidative stress in the brain of rats exposed in utero to cocaine. Ann. N. Y. Acad. Sci..

[14] Nassogne MC, Louahed J, Evrard P, Courtoy PJ (1997). Cocaine induces apoptosis in cortical neurons of fetal mice. J. Neurochem..

[15] Poon HF, Abdullah L, Mullan MA, Mullan MJ, Crawford FC (2007). Cocaine-induced oxidative stress precedes cell death in human neuronal progenitor cells. Neurochem. Int..

[16] Numa R, Kohen R, Poltyrev T, Yaka R (2008). Tempol diminishes cocaine-induced oxidative damage and attenuates the development and expression of behavioral sensitization. Neuroscience.

[17] Pomierny-Chamioło L, Moniczewski A, Wydra K, Suder A, Filip M (2013). Oxidative stress biomarkers in some rat brain structures and peripheral organs underwent cocaine. Neurotox. Res..

[18] Lipton JW (2003). Prenatal cocaine administration increases glutathione and alpha-tocopherol oxidation in fetal rat brain. Dev. Brain Res..

[19] Macêdo DS (2005). Cocaine alters catalase activity in prefrontal cortex and striatum of mice. Neurosci. Lett..

[20] Walker J (2014). Total antioxidant capacity is signi fi cantly lower in cocaine-dependent and methamphetamine-dependent patients relative to normal controls: results from a preliminary study. Hum. Psychopharmacol Clin Exp.

[21] Muriach M (2010). Cocaine causes memory and learning impairments in rats: Involvement of nuclear factor kappa B and oxidative stress, and prevention by topiramate. J. Neurochem..

[22] Sordi, A. O. rgle *et al*. Oxidative stress and BDNF as possible markers for the severity of crack cocaine use in early withdrawal. *Psychopharmacology (Berl)*. **231**, 4031–4039 (2014). 10.1007/s00213-014-3542-1 24676990

[23] Cuzzocrea S (2000). Effects of tempol, a membrane-permeable radical scavenger, in a gerbil model of brain injury. Brain Res..

[24] Thiemermann C (2003). Membrane-permeable radical scavengers (tempol) for shock, ischemia-reperfusion injury, and inflammation. Crit Care Med..

[25] Trembovler V (1999). Antioxidants attenuate acute toxicity of tumor necrosis factor-alpha induced by brain injury in rat. J. Interferon Cytokine Res..

[26] Samuni A, Krishna CM, Riesz P, Finkelstein E, Rusw A (1988). A Novel Metal-free Low Molecular Weight Superoxide Dismutase Mimic. J. Biol. Chem..

[27] Numa R, Baron M, Kohen R, Yaka R (2011). Tempol attenuates cocaine-induced death of PC12 cells through decreased oxidative damage. Eur. J. Pharmacol..

[28] Jang EY (2015). Involvement of reactive oxygen species in cocaine-taking behaviors in rats. Addict. Biol..

[29] Jang, E. Y. *et al*. The role of reactive oxygen species in methamphetamine self-administration and dopamine release in the nucleus accumbens. *Addict. Biol*. doi:10.1111/adb.12419 (2016). 10.1111/adb.12419 PMC5237425 27417190

[30] Qi C (2015). mGluR5 in the nucleus accumbens shell regulates morphine-associated contextual memory through reactive oxygen species signaling. Addict. Biol..

[31] Misko TP, Schilling RJ, Salvemini D, Moore WM, Currie MG (1993). A Fluorometric Assay for the Measurement of Nitrite in Biological Samples. Analytical Biochemistry.

[32] Kamatari M, Yasui H, Ogata T, Sakurai H (2002). Local pharmacokinetic analysis of a stable spin probe in mice by *in vivo* L-band ESR with surface-coil-type resonators. Free Radic. Res..

[33] Soule BP (2007). The chemistry and biology of nitroxide compounds. Free Radic. Biol. Med..

[34] De Macedo IC, De Freitas JS, Da Silva Torres IL (2016). The influence of palatable diets in reward system activation: A mini review. Advances in Pharmacological Sciences.

[35] Malenka RC, Kauer J (2007). a. Synaptic plasticity and addiction. Nat. Rev. Neurosci..

[36] Ungless MA, Whistler JL, Malenka RC, Bonci A (2001). Single cocaine exposure *in vivo* induces long-term potentiation in dopamine neurons. Nature.

[37] Borgland SL, Malenka RC, Bonci A (2004). Acute and chronic cocaine-induced potentiation of synaptic strength in the ventral tegmental area: electrophysiological and behavioral correlates in individual rats. J. Neurosci..

[38] Thomas MJ, Beurrier C, Bonci A, Malenka RC (2001). Long-term depression in the nucleus accumbens: a neural correlate of behavioral sensitization to cocaine. Nat. Neurosci..

[39] Conrad KL (2008). Formation of accumbens GluR2-lacking AMPA receptors mediates incubation of cocaine craving. Nature.

[40] Schumann J, Yaka R (2009). Prolonged withdrawal from repeated noncontingent cocaine exposure increases NMDA receptor expression and ERK activity in the nucleus accumbens. J. Neurosci..

[41] Itzhak Y, Anderson KL (2007). Memory Reconsolidation of CocaineAssociated Context Requires Nitric Oxide Signaling. Synapse.

[42] Selvakumar B (2013). S-nitrosylation of AMPA receptor GluA1 regulates phosphorylation, single-channel conductance, and endocytosis. Proc. Natl. Acad. Sci. U. S. A..

[43] Hermida-Ameijeiras Á, Méndez-Álvarez E, Sánchez-Iglesias S, Sanmartín-Suárez C, Soto-Otero R (2004). Autoxidation and MAO-mediated metabolism of dopamine as a potential cause of oxidative stress: Role of ferrous and ferric ions. Neurochem. Int..

[44] Kloss MW, Rosen GM, Rauckman EJ (1984). Biotransformation of Norcocaine to Norcocaine Nitroxide by Rat-Brain Microsomes. Psychopharmacol..

